# Cellular mechanisms underlying state-dependent neural inhibition with magnetic stimulation

**DOI:** 10.1038/s41598-022-16494-8

**Published:** 2022-07-15

**Authors:** Hui Ye, Vincent Chen, Jenna Hendee

**Affiliations:** 1 Department of Biology, Quinlan Life Sciences Education and Research Center, Loyola University Chicago, 1032 W. Sheridan Rd., Chicago, IL 60660 USA; 2 Engineering Program, Loyola University Chicago, Chicago, IL USA

**Keywords:** Membrane biophysics, Molecular biophysics, Neuroscience, Physiology, Mathematics and computing

## Abstract

Novel stimulation protocols for neuromodulation with magnetic fields are explored in clinical and laboratory settings. Recent evidence suggests that the activation state of the nervous system plays a significant role in the outcome of magnetic stimulation, but the underlying cellular and molecular mechanisms of state-dependency have not been completely investigated. We recently reported that high frequency magnetic stimulation could inhibit neural activity when the neuron was in a low active state. In this paper, we investigate state-dependent neural modulation by applying a magnetic field to single neurons, using the novel micro-coil technology. High frequency magnetic stimulation suppressed single neuron activity in a state-dependent manner. It inhibited neurons in slow-firing states, but spared neurons from fast-firing states, when the same magnetic stimuli were applied. Using a multi-compartment NEURON model, we found that dynamics of voltage-dependent sodium and potassium channels were significantly altered by the magnetic stimulation in the slow-firing neurons, but not in the fast-firing neurons. Variability in neural activity should be monitored and explored to optimize the outcome of magnetic stimulation in basic laboratory research and clinical practice. If selective stimulation can be programmed to match the appropriate neural state, prosthetic implants and brain-machine interfaces can be designed based on these concepts to achieve optimal results.

## Introduction

Magnetic stimulation on excitable biological tissues was first reported in the early twentieth century by Jacques d’Arsonval (1896) and Thompson (1910) with their work on human visual sensations. Modern development of novel stimulation protocols with magnetic field have been explored in clinical and laboratory environments for effective neuromodulation and treatment of neurological diseases, such as epilepsy^1,2^. Control of neural activity with the magnetic field is largely dependent on the stimulation parameters, such as duration, frequency, intensity, and orientation of the magnetic coil to the targeted neurons^3^. In clinical practice, each of these parameters have been carefully optimized to produce the best outcome with transcranial magnetic stimulation (TMS)^4–6^. In basic lab research, these parameters are associated with the excitation of individual neurons^7^, alternation of synaptic transmission^8,9^, and changes in ion channel dynamics^10^.

Besides the parameters that define the magnetic stimuli, emerging evidence has strongly suggested that biophysical properties of the neural tissue could also affect the outcome of magnetic stimulation. Tissue heterogeneity and anisotropy significantly alter the magnetically induced electric field and current density distribution in the brain^11–13^. At the single cell level, cells of different classes respond to the same magnetic stimuli differently^14^. The cumulative effects of magnetic stimulation depend on the structure of the targeted neural circuit, morphology, and electric properties of the neurons^3^.

Recent evidence further suggests that the excitation state of the nervous system might play a significant role in the outcome of magnetic stimulation (termed “state-dependent”). For example, magnetic stimulation produces different perceptual or behavioral outcomes that may depend on the excitability levels of specific neuronal populations^15^. It has been found that the instantaneous brain state can be used to promote efficacious plasticity induction by TMS^16^. Recordings of extracellular spikes and local field potentials from the cat visual cortex following TMS have demonstrated that responses to TMS depend on the state of neural activity^17^. The presence of electric discharge (measured by spikes and local field potential) in the visual cortex after TMS depends on the pre-TMS activity in the recorded area. These observations suggest that the stimulation effects of magnetic fields could be dependent on the active state of individual neurons. However, this possibility has never been thoroughly studied with intracellular technology, and the ion channel mechanisms underlying this phenomenon are largely unknown.

To address if the excitation state of the neuron could indeed affect the outcomes of magnetic stimulation, one would need an experimental system that allows the control and monitoring of an individual neuron’s activity under magnetic stimulation. Such an experimental design is technically challenging since the noise induced by time-varying magnetic stimulation could interfere with and contaminate the intracellular recording. Furthermore, the conventional large coil used in clinical settings could not provide the level of specificity required in such applications.

The large neurons and long nerve projections in the buccal ganglion in the marine mollusk, *Aplysia californica,* provide an ideal system for the study of electric^18^ and magnetic stimulation^19^ at the single cell level. Using the buccal ganglion neurons, we^20^ recently reported that high frequency magnetic stimulation with a micro-coil could inhibit neural activity. In this published study, the firing frequency of the neurons was relatively low, and the inhibitory effects of the magnetic field were significant. Neurons were instantly and completely inhibited when the miniature coil was turned on to apply high frequency (i.e., 400 Hz) stimulation.

In this paper, we test the hypothesis that neural inhibition by magnetic stimulation is dependent on the activation state of the targeted neurons. We used several in vitro protocols to drive the neuron to fire action potentials at various frequencies. The state of the neuron is, therefore, defined as the frequency of action potentials in the neuron. We found that when the neuron was at a low firing state, the magnetic stimulation protocol was effective in inhibiting the neuron. In contrast, when the neuron was at a high firing state, the same magnetic stimulation became incapable of blocking the somatic activation in the neuron.

To further investigate the cellular and ion channel mechanisms underlying “state-dependent” magnetic blockage, we further developed the computer simulation methods used in the previous work^20^. We directly measured the magnetically induced electric field and incorporated this new information into the biophysical modeling and NEURON simulation work. We believe these new steps made our modeling endeavor significantly closer to the biological reality. Using the improved NEURON model, we provide a mechanistic explanation of the state-dependency in single neuron inhibition by the magnetic field.

## Results

### Magnetic inhibition of *spontaneous* neural activity is state-dependent

**Figure 1:**
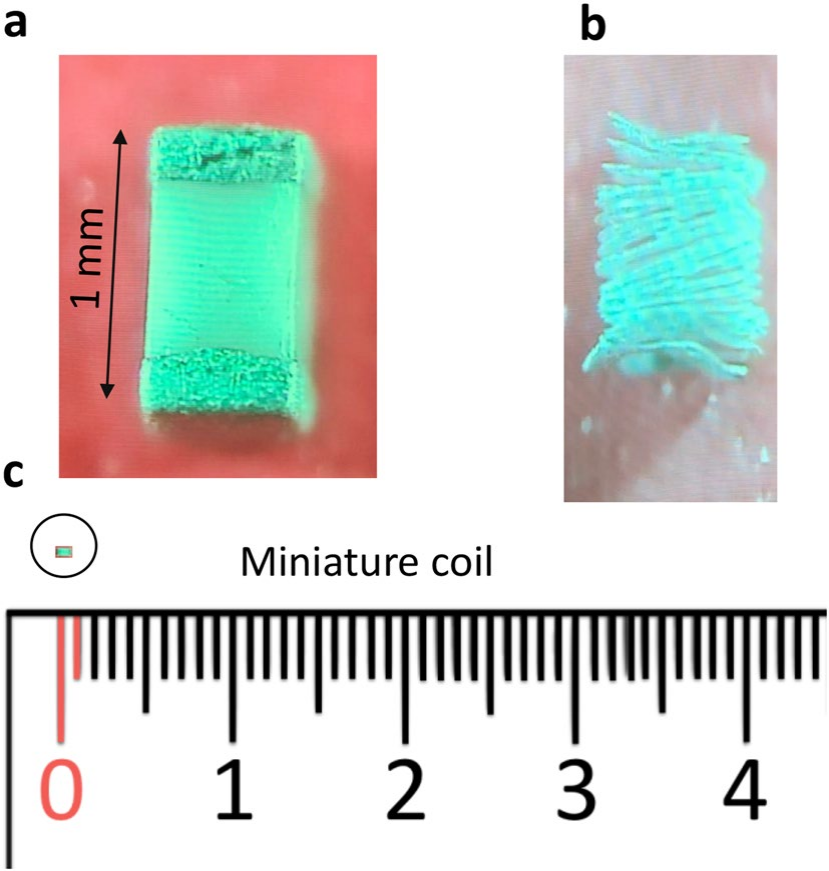
The miniature coil used for the magnetic stimulations. (**a**) The inductor with a size of 1 mm × 0.5 mm × 0.5 mm. (**b**) The internal structure of the coil was revealed by removing the ceramic cover with chemicals. The structure of the coil included 20 loops in rectangular shape. (**c**) The size of the coil was compared to a ruler for reference.

To investigate the effects of magnetic stimulation on the individual neurons, we used the novel technology of miniature coil stimulation, which provides focal neural stimulation. A commercial multilayer surface mount inductor was selected for the study thanks to its small size (1 mm × 0.5 mm × 0.5 mm) and capability of producing a large electric field^20,21^. To further reveal the internal structure of the coil, we chemically dissolved the coil encapsulation (Fig. 1). Each inductor contains 20 loops of rectangular shape.

**Figure 2:**
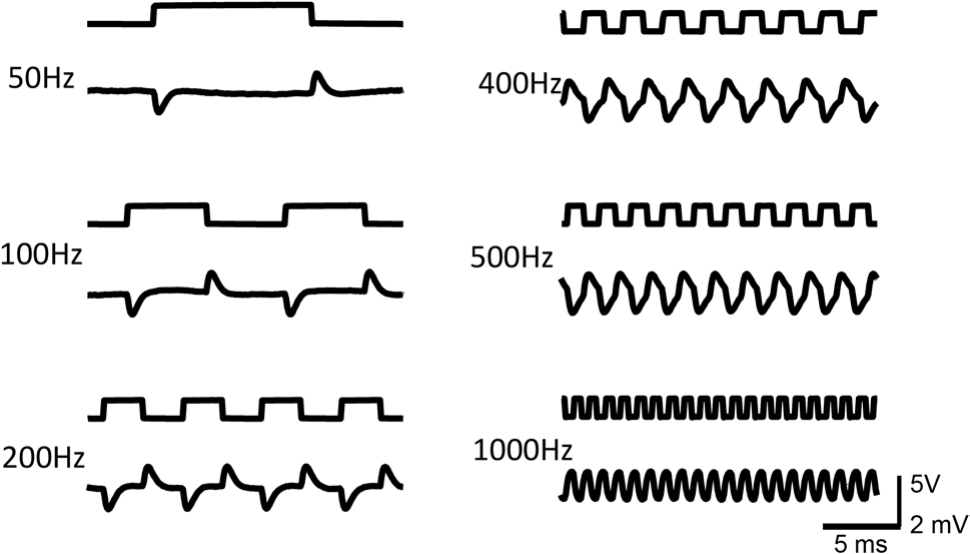
Input voltage to the coil and the magnetically induced electric potential. Upper traces: Square waves (10 V) of various frequencies were generated by a signal generator and were delivered to the miniature coil via a power amplifier. Lower traces: Magnetically induced electric potential recorded in the petri dish.

The coil was driven by a power amplifier to produce high frequency stimulation. Monophasic square pulses of various frequencies were generated by a signal generator and delivered to the power-amplifier. To estimate the waveform of the induced electric field, we measured it close to the coil in the petri dish. Consistent with previous reports^22,23^, the miniature coil generated electric voltages in a biphasic shape (Fig. 2), suggesting that the neurons will be stimulated only during the rising and falling phases of each pulse.

**Figure 3:**
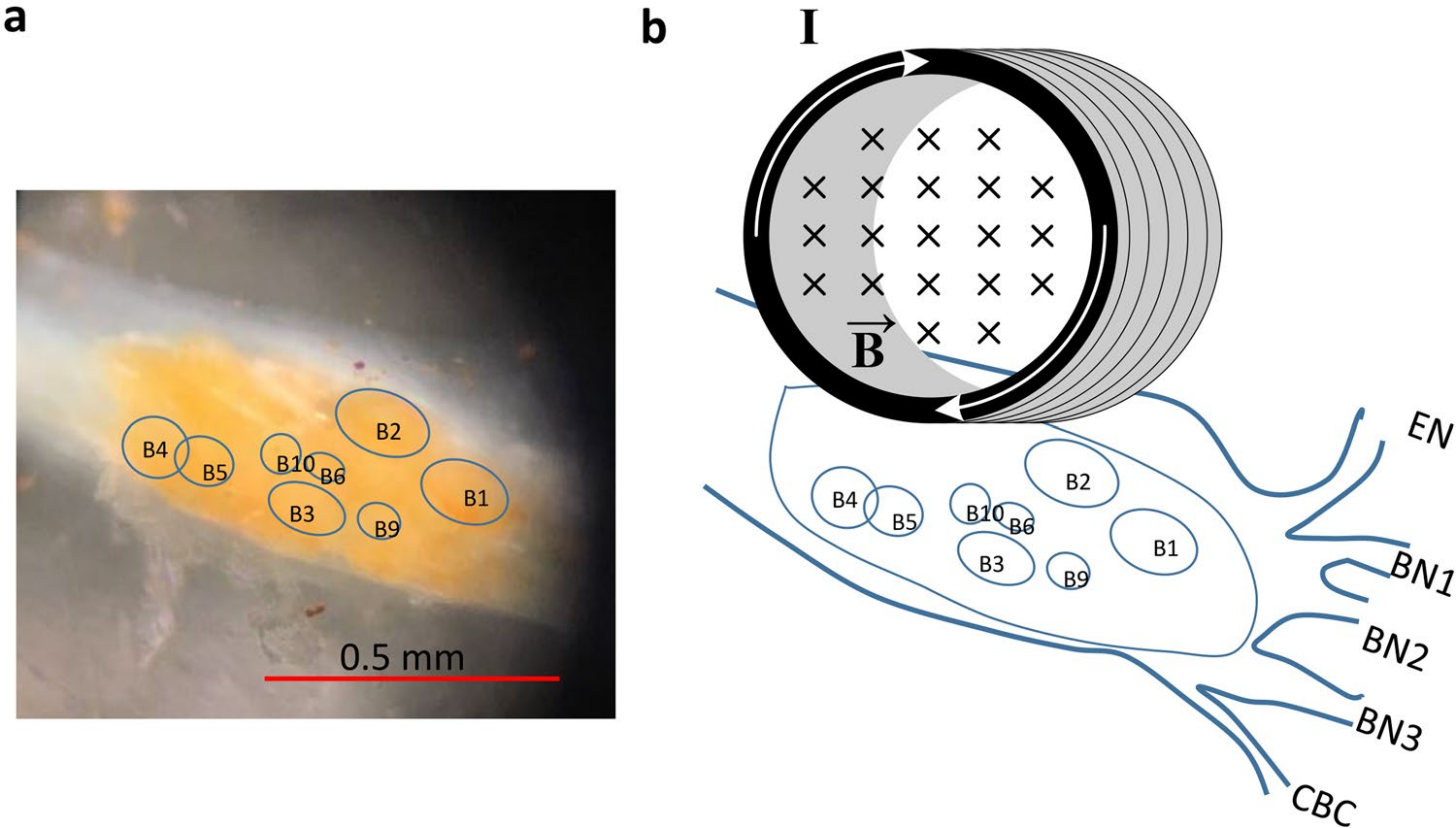
Magnetic stimulation applied to the buccal ganglion of *Aplysia*. (**a**) The buccal ganglion and the identified neurons. (**b**) An illustration of the buccal ganglion under miniature coil stimulation. A miniature coil was positioned on top of the buccal ganglion for magnetic stimulation (I: electric current inside the coil, B: inward magnetic field generated by the electric current). Locations of interneurons (B4 and B5), motor neurons (B3, B6, B9, and B10), and several other neurons and nerves (EN: esophageal nerve, BN1: buccal nerve I, BN2: buccal nerve II, BN3: buccal nerve III, and CBC: cerebro-buccal connection) were identified in the illustration.

The miniature coil was positioned carefully above the buccal ganglion for stimulation (Fig. 3). For miniature coil stimulation, orientation of the miniature coil to the cell has been shown to play significant roles in axon stimulation^24^. Previously, it has been shown that efficient neural stimulation requires the induced electric field be parallel to the soma-axon axial^25,26^. We positioned the coil so that the coil loop was in parallel to the buccal ganglion—BN2 axial (Fig. 3b). This ensured that the induced electric field had the largest gradient along the soma-axon axial to produce efficient stimulation^20^.

To investigate state-dependent inhibition of single neuron activity by the miniature coil, we intracellularly recorded from several jaw motor neurons (B3, B6, and B9) in the buccal ganglion of *Aplysia*. These large neurons have similar physiological properties. Morphologically, they are located within a small area on the caudal surface of the buccal ganglion. They all innervate the I1/I3 muscle by sending axons through the buccal nerve II (BN2). Activation of these neurons is responsible for jaw closure during the feeding behavior of *Aplysia*^27–29^. Therefore, these neurons are likely stimulated by the same magnetic intensity.

**Figure 4:**
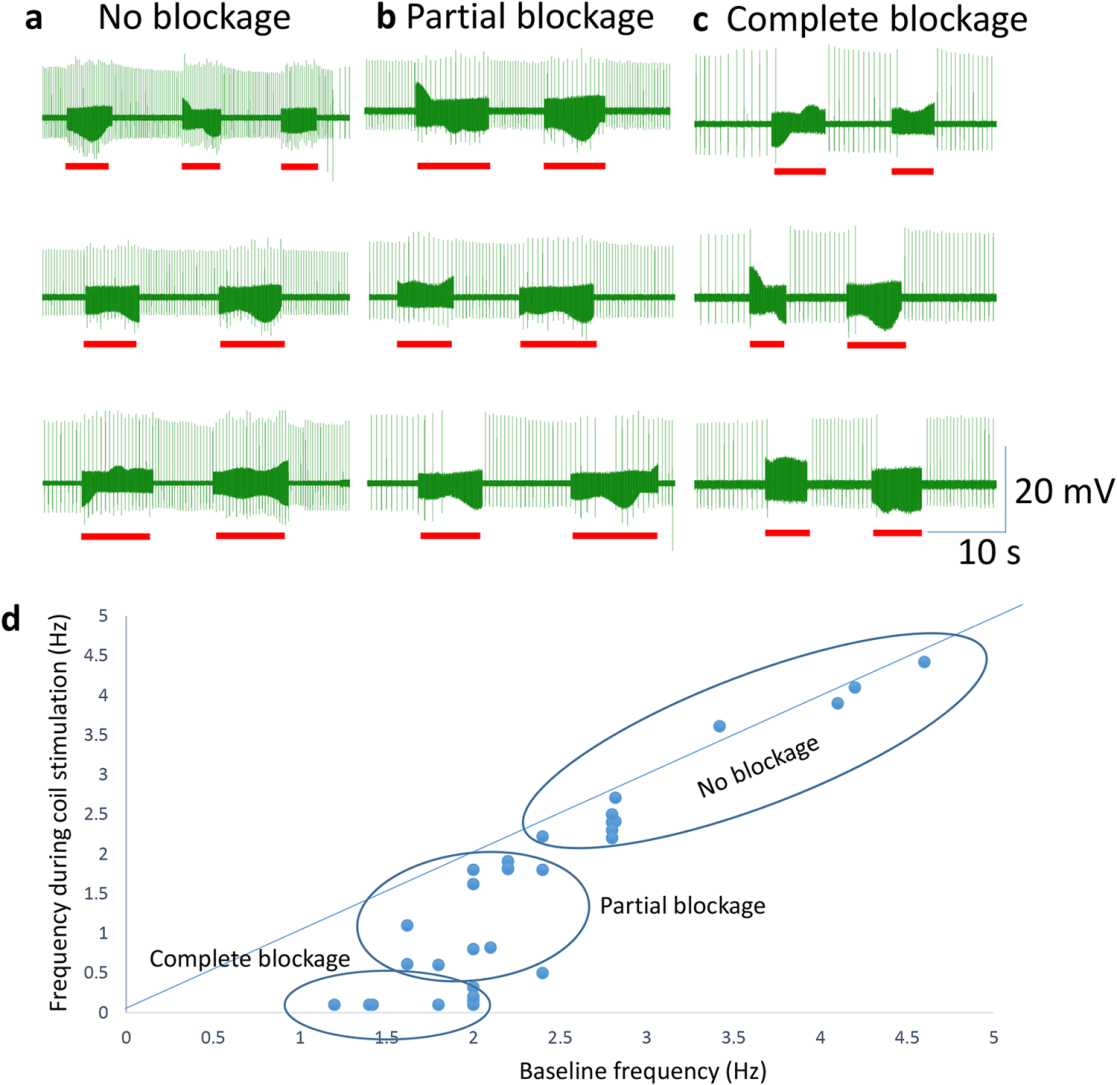
Magnetic blockage of neural activity was dependent on the spontaneous firing frequency in the neuron. (**a**) Magnetic stimulation (400 Hz, red bars) was ineffective when the neuron exhibited high firing rates. (**b**) Magnetic stimulation partially blocked the neuron that was moderately active. (**c**) Magnetic stimulation completely suppressed the neuron whose activity was low. In (**a**–**c**), three representative traces for the neuron were shown at each activation state. (**d**) Firing frequency of the neuron in baseline recording versus that during magnetic stimulation.

When the sharp electrode was inserted into the soma, it recorded spontaneous activity in the neuron, which usually lasted for 10–20 min, until the neuron became quiescent again. We tested the capability of magnetic stimulation in inhibiting these action potentials. We applied 400 Hz square waves to the coil for several seconds. When the neuron fired at a higher frequency (> 3 Hz), the coil was incapable of inhibiting neural activity (Fig. 4a). In contrast, when the neuron’s firing frequency was relatively low (1.5–3 Hz), coil stimulation could further decrease the intrinsic firing frequency (Fig. 4b). When the neuron fired at a lower frequency (< 1.5 Hz), the coil could reversibly and completely block neural activity (Fig. 4c).

To demonstrate quantitatively the frequency-dependency of coil inhibition in neural activity, we plotted the firing frequency of the neuron during the coil stimulation against its baseline firing frequency right before the stimulation (Fig. 4d, n = 31). This scatter plot demonstrates that high frequency magnetic stimulation could partially or completely block neural activity, depending on the intrinsic frequency of the neuron. In this plot, the diagonal line indicates there is no significant difference in the neuron’s firing frequency before and during coil stimulation. State-dependent magnetic inhibition was observed in all five motor neurons tested under this protocol.

**Figure 5:**
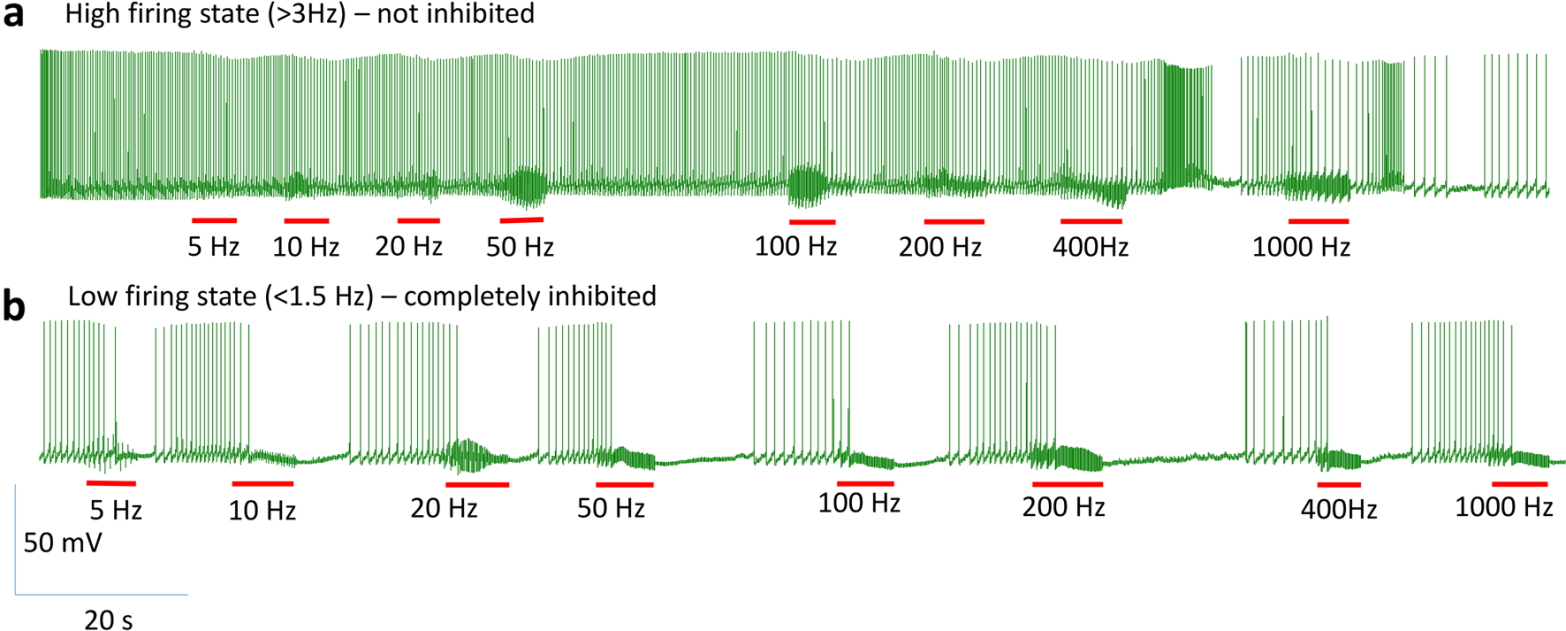
State-dependent soma inhibition by the miniature coil with a range of stimulation frequencies. Neural activity in a spontaneously firing neuron was recorded. Stimuli (red bars) ranging in frequency (5–1000 Hz) were applied to the coil. (**a**) Magnetic stimulation had a minimal effect when the neuron was at a high firing state (> 3 Hz). (**b**) Complete magnetic inhibition was observed for all stimulation frequencies when the neuron was at a low firing state (< 1.5 Hz).

State-dependent inhibition of neuron activity was also observed when the frequency of the stimulus varied. We applied a spectrum of stimulation frequencies to the coil (5–1000 Hz). When the recorded neuron was at a highly active state (> 3 Hz), magnetic stimulation failed to completely block the neural activity (Fig. 5a). In contrast, when the cell was at a relatively low active state (< 1.5 Hz), all stimuli could completely block neuron activity (Fig. 5b). State-dependent magnetic inhibition was observed in all 5 motor neurons tested under this protocol, when spontaneous neural activity was present.

### Magnetic inhibition of induced neural activity is state-dependent

Since the level of neural activity is essential in the neuronal response to magnetic inhibition, in the following experiments, the firing frequency of the neuron was deliberately controlled.

**Figure 6:**
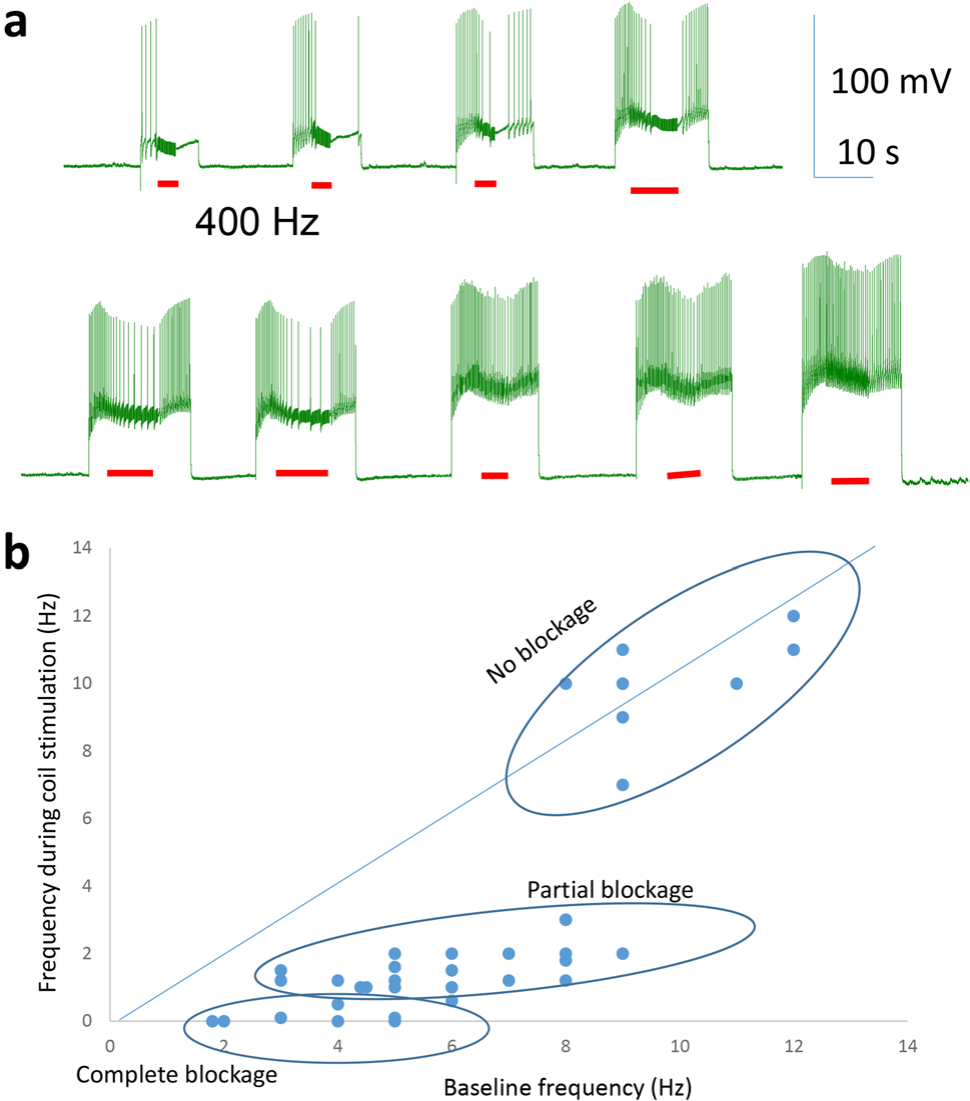
Magnetic inhibition was dependent on the level of neuronal activity induced by sustained depolarization current. (**a**) Depolarization currents with gradual increases in intensity (starting from 1 to 9 nA, with 1 nA increments) were injected into the neuron (resting potential − 55 mV) to elicit action potentials. Magnetic stimulation (400 Hz, red bars) was applied to the soma for inhibition. When firing with low frequency, the neuron was completely inhibited by the miniature coil. When firing at a high frequency, the neuron sustained its activity during magnetic stimulation. (**b**) Firing frequency of the neuron in baseline recording versus that during magnetic stimulation.

In the first protocol, constant electric currents were injected into the quiescent motor neurons for about 15 s. The depolarization current was gradually increased to elicit more activity in the neuron. This caused the neuron to fire action potentials at different frequencies (Fig. 6a). A 400 Hz stimulation (approximately 5–10 s in duration) was then applied to this cell for neural blockage. Consistent with our previous study^20^, complete inhibition was observed when the neuron fired between 2 and 5 Hz (Fig. 6a). In contrast, neurons firing between 3 and 8 Hz were partially inhibited. If the neuron fired at a high frequency (above 8 Hz), its activity was not affected by the coil stimulation.

We quantified the total 36 trials and plotted the firing frequency during magnetic stimulation against the frequency immediately before stimulation (Fig. 6b). State-dependent magnetic inhibition was observed in all five motor neurons tested under this protocol.

**Figure 7:**
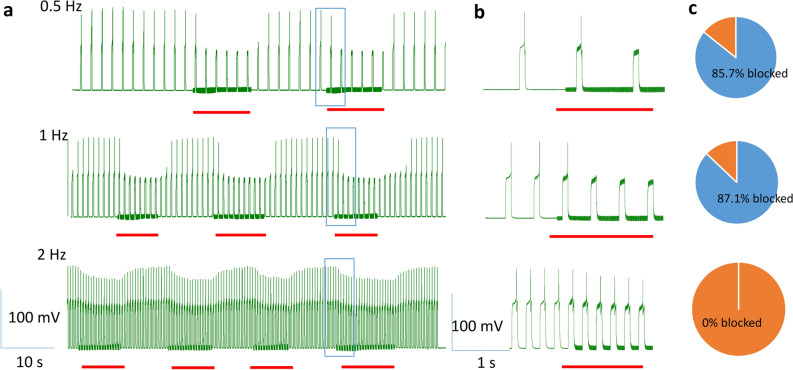
Magnetic inhibition is dependent on the level of neuronal activity induced by pulse stimulation. The neuron was elicited to fire a single action potential by short current pulses at 0.5, 1, and 2 Hz, respectively. The coil stimulated the soma at 400 Hz for approximately 10 s (red bars). (**a**) Magnetic stimulation inhibited the soma firing at a low rate (0.5 and 1 Hz) but did not block the soma firing at a higher rate (2 Hz). (**b**) Expanded traces of the blue rectangles in (a). (**c**) Percentage of successful blockage by the magnetic field in multiple trials.

In the second protocol, we delivered short current pulses to the neuron at a fixed frequency: 0.5, 1, or 2 Hz (Fig. 7). Duration of the pulses was adjusted so that each electric pulse could trigger one single action potential. The coil stimulated the soma at 400 Hz for approximately 10 s (red bars). Consistent with our previous study^20^, the magnetic stimulation inhibited the neuron when it was driven to fire at a low rate (0.5 and 1 Hz) that was triggered by the short pulses. In contrast, the magnetic stimulation did not block the soma firing at a high rate (2 Hz, Fig. 7b). Statistically, at 0.5 Hz firing rate, 30/35 action potentials were blocked; at 1 Hz firing rate, 54/62 action potentials were blocked. These measures are not statistically different (${x}^{2}$=0.037, *p* = 0.85). When the neurons fired at 2 Hz, 0/112 action potentials were blocked by the magnetic stimulation (Fig. 7c). This rate of inhibition at 2 Hz was significantly lower than the recorded neurons firing at 0.5 Hz (${x}^{2}$=111.94, *p* < 0.001) and at 1 Hz (${x}^{2}$=132.45, *p* < 0.001). State-dependent magnetic inhibition was observed in all five motor neurons tested under this protocol.

In summary, coil stimulation successfully blocked the neurons in a slow-firing state, but spared neuron in a fast-firing state.

### Computational simulation confirmed that magnetic inhibition of neuron activity is state-dependent

**Figure 8:**
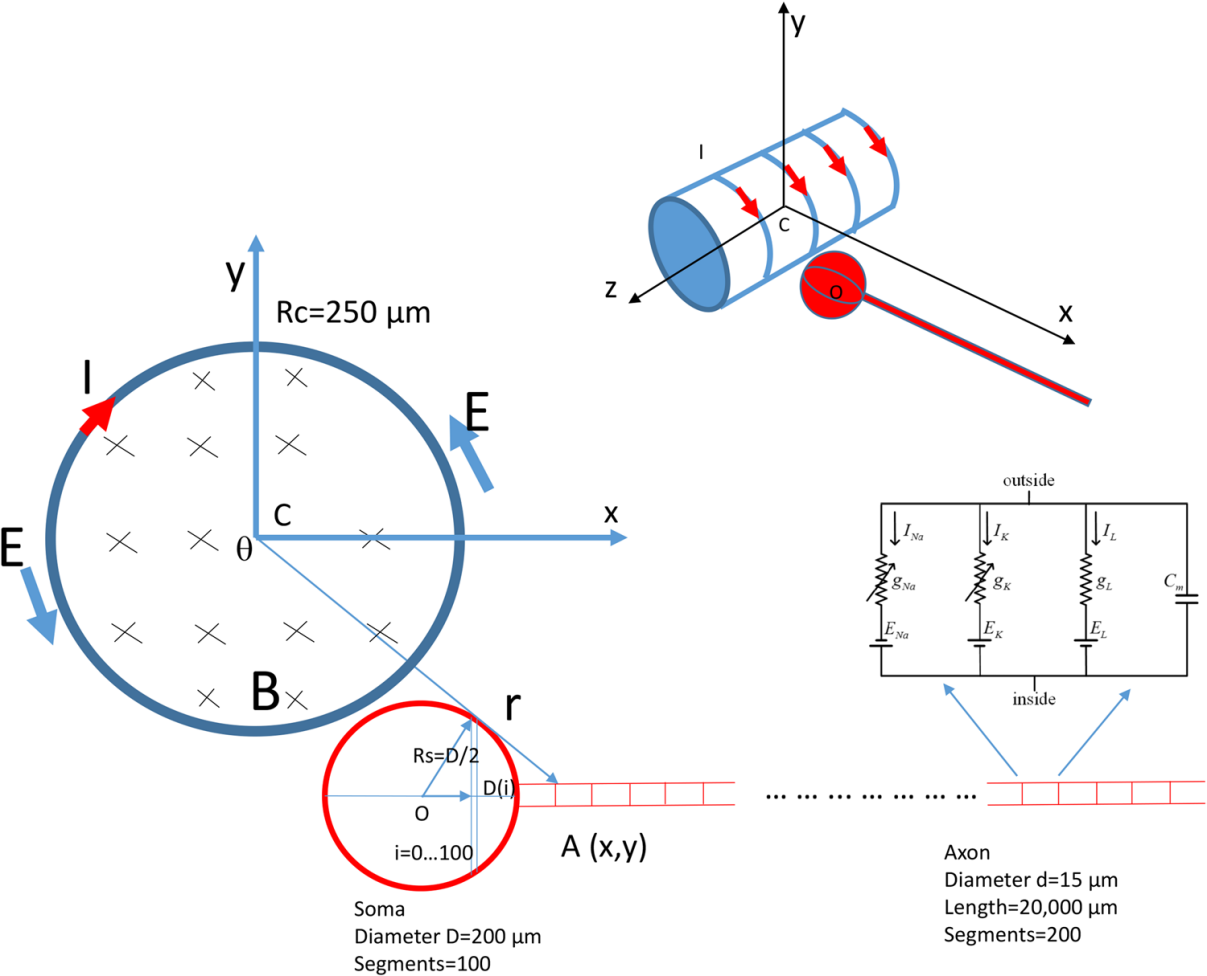
Multi-compartment neuron model under magnetic stimulation. The neuron was stimulated by a cylindrical coil (radius 250 μm), whose axis overlapped with the z-axis. High frequency square pulses were delivered to the coil to induce the electric field (E: electric field, I: coil current, and B: inward magnetic field generated by the electric current). The modeled neuron included a spherical soma (200 μm in diameter, 100 segments) and a cylindrical axon (15 μm in diameter, 20,000 μm in length, 200 segments). Each neural compartment was inserted with Hodgkin-Huxley type ion channels. The center of the soma (O) is located close to the center of the coil (C), and the axon was in the x direction. Point A (x, 0) was a point on the neuron, whose distance to the center of the coil is r.

To simulate soma inhibition by the high frequency magnetic stimulation, we utilized a modified version of our published biophysical model that computed the magnetically induced electric field in the vicinity of the neuron^20^. The modified coil model includes the temporal profile of the induced electric field, which was validated by direct measurement (Fig. 2). We then applied this modified electric field to a multi-compartment model of the *Aplysia* neuron (Fig. 8).

To simulate the different activation states of the neuron, we injected different levels of depolarization currents to the modeled neuron to elicit action potentials of various frequencies. We applied 400 Hz stimulation pulses to the coil, as in the electrophysiological experiments.

**Figure 9:**
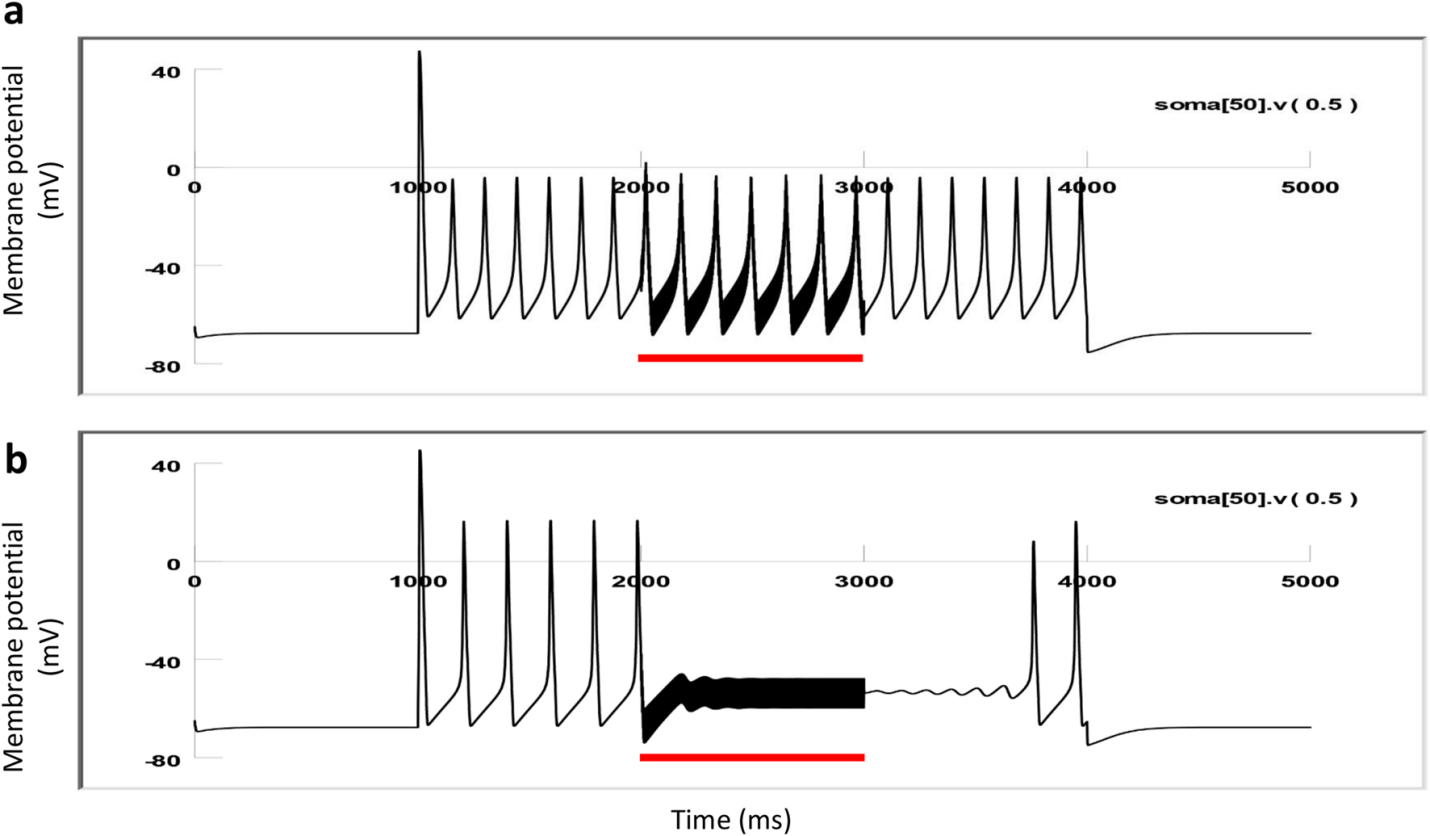
Neural inhibition by the magnetic field is dependent on the active state of the neuron. 400 Hz coil stimulation (red lines) was applied to the modeled neuron. (**a**) 35 nA depolarization current elicited high firing frequency (7 Hz) and the magnetic stimulation failed to inhibit the neuronal activity. (**b**) 20 nA depolarization current elicited moderate firing (5 Hz) and the magnetic stimulation inhibited neural activity instantly.

Simulation confirmed that the high frequency magnetic pulses could inhibit the neuron, and the inhibitory effects were dependent on the firing frequency of the neuron. In Fig. 9a, a depolarization current (35 nA) was injected into the soma to trigger action potentials at a high firing rate (8 Hz) for 3000 ms. When the firing of the neuron was steady, we applied a 1000 ms pulse train (400 Hz) to the soma. The magnetic stimulation caused some fluctuation of the membrane potential, but it failed in blocking the action potentials.

In contrast, when the neuron was firing at a relatively low frequency, coil stimulation became effective in neural suppression. In Fig. 9b, a smaller depolarization current (20 nA) was injected into the soma to trigger action potentials with much lower frequency (5–6 Hz) than in Fig. 9a. When the firing of the neuron became steady, we applied the same 1000 ms pulse train (400 Hz) to the soma. The stimulation pulses instantly blocked the action potentials. These simulation results replicate the electrophysiological data in Fig. 6, in which neurons were injected with a long depolarization current to trigger action potentials. Withdrawal of the coil stimulation allowed the neuron to resume its firing capability to the level before stimulation. In this simulation, the miniature coil was positioned close to the soma for specific stimulation (Fig. 3). Varying the coil center could cause changes in the distance between the targeted neuron and the coil. By adjusting the stimulation intensity, the coil could consistently block the neuron’s firing in a state-dependent manner.

### Computational simulation revealed ion channel mechanisms underlying the state-dependent magnetic inhibition of neural activity.

Previous studies have demonstrated that electric or magnetic stimulation could affect ion channel functions. Among these channels, voltage-dependent sodium channels and potassium channels are the most studied since they directly contribute to the initiation and sustainability of the action potentials. For example, high frequency stimulation using monophasic electric current was shown to depolarize the membrane, inactivate sodium channels, and impair the mechanisms of neuronal firing^30^. Low frequency magnetic simulation altered the kinematics of sodium and potassium channels in the hippocampal pyramidal neurons^31^.

To investigate the ionic mechanisms underlying magnetic inhibition and its state-dependency, we monitored the inward sodium current (INa^+^) and the outward potassium current (IK^+^) during magnetic stimulation. To illustrate the kinetics of the sodium channel, we plotted the activation (m) and inactivation (h) variables for the sodium channels. To illustrate the kinetics of the potassium channels, we plotted the activation variable (n) for the potassium channels during NEURON simulation of coil stimulation.

**Figure 10:**
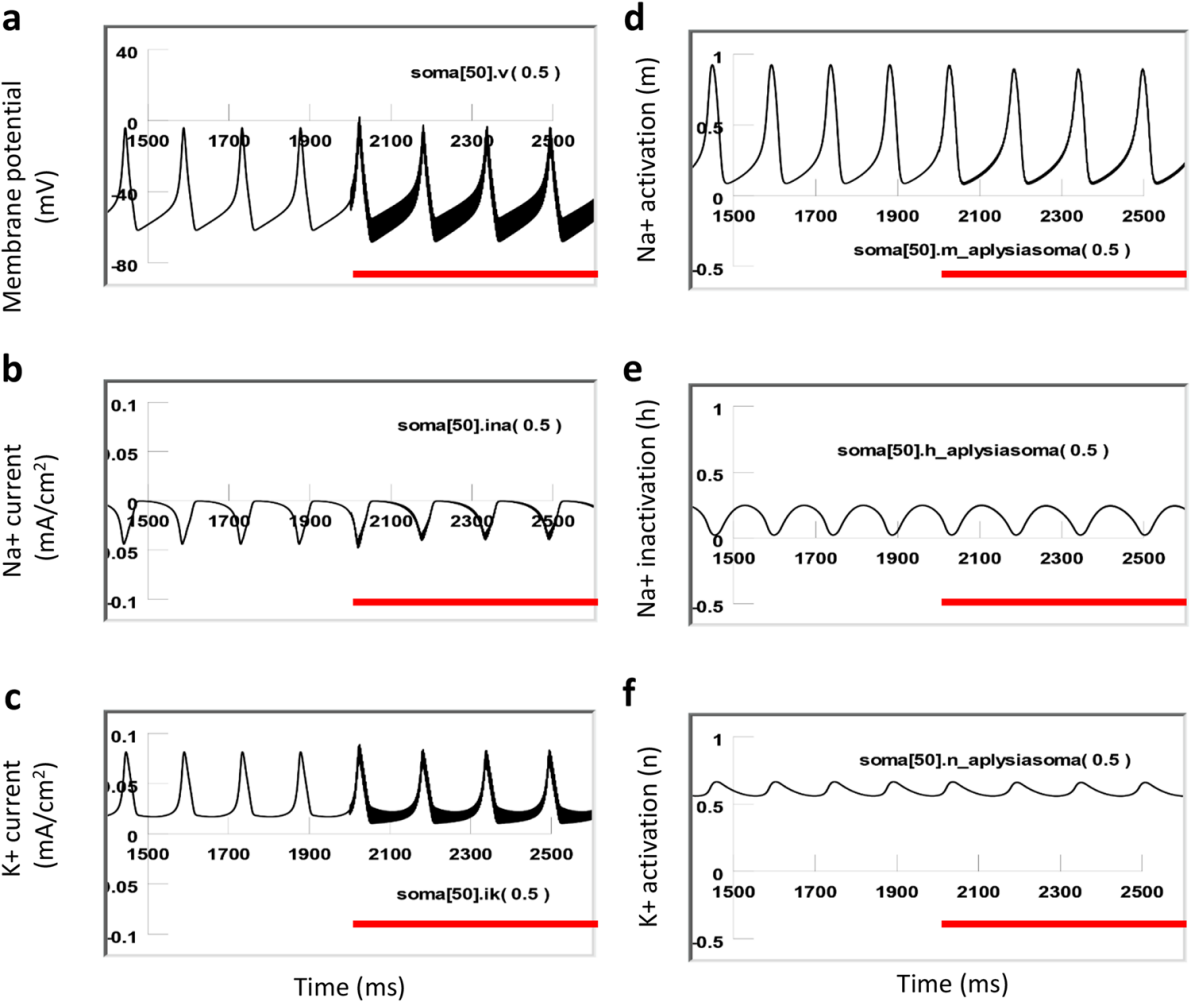
Ion channel dynamics of a highly active neuron (7 Hz) under magnetic stimulation with the miniature coil (red lines). (**a**) Membrane potential. (**b**) Na^+^ current. (**c**) K^+^ current. (**d**) Na^+^ channel activation (m). (**e**) Na^+^ channel inactivation (h). (**f**) K^+^ channel activation (n).

**Figure 11:**
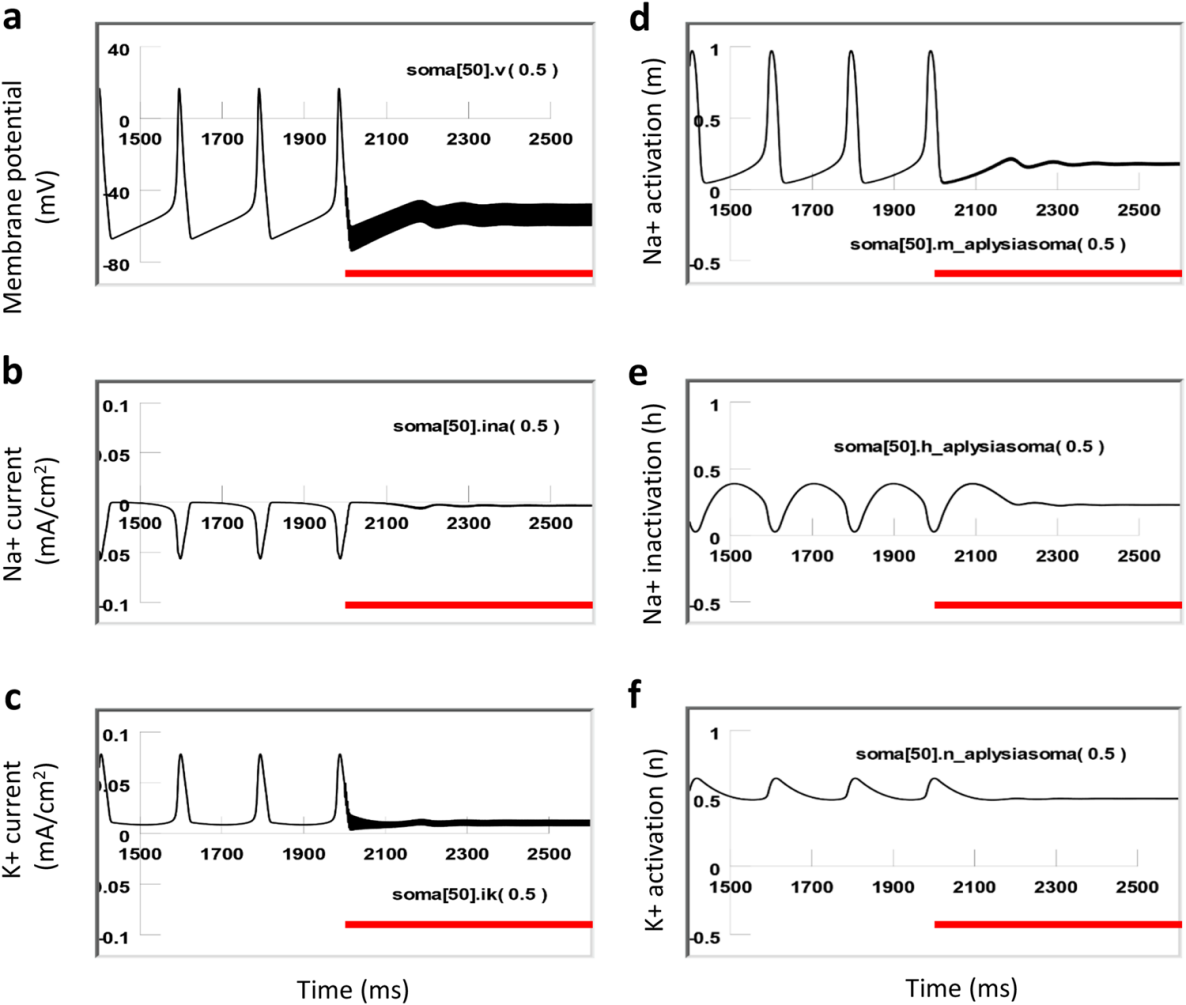
Ion channel dynamics of a relatively low-active neuron (5 Hz) under magnetic stimulation (red lines) with the miniature coil. (**a**) Membrane potential. (**b**) Na^+^ current. (**c**) K^+^ current. (**d**) Na^+^ channel activation (m). (**e**) Na^+^ channel inactivation (h). (**f**) K^+^ channel activation (n).

In the absence of coil stimulation, the membrane was at resting potential (− 65 mV). Depolarization currents were applied to the soma to drive neuronal firing at different frequencies. Strong depolarization of the soma elicited constant firing of the soma at a higher frequency (8 Hz, Fig. 10a). Under weak depolarization, the soma fired at a moderate frequency (5 Hz, Fig. 11a). In both cases, the sodium channel was modestly de-inactivated (h = 0.3 in Fig. 10e, and h = 0.4 in Fig. 11e) before the firing of each action potential. This allowed a sufficient activation of the sodium channels (m = 0.95, Figs. 10d, 11d) to produce a large inward sodium current (INa^+^, Figs. 10b, 11b) and depolarization of the membrane for spiking. Meanwhile, activation of the potassium channels was substantial (n = 0.65, Figs. 10f, 11f), and a large inward potassium current was observed to hyperpolarize the membrane during the falling phase of the action potentials (Figs. 10c, 11c).

When a neuron of relatively high activity (8 Hz) was stimulated by the magnetic coil, there was a fast oscillation in the membrane potential, due to the oscillatory nature of the magnetically induced electric field. This oscillatory effect can also be observed in the INa^+^ and IK^+^ current traces, defined by the product of the driving force (the difference between the membrane potential and the equilibrium potential of a specific ion) and the conductance of the individual ion channels. Because of the voltage-dependency of the state variables (m, h, and n), small oscillatory effects were also observed on state variable traces (Fig. 10d–f). However, the overall kinetics of the state variables were not altered by the magnetic stimulation. The highly active neuron was able to sustain a fast, inward INa^+^ and a delayed, long IK^+^, and generate normal action potentials during the magnetic stimulation.

In contrast, when a neuron of low or moderate activity (5–6 Hz) was stimulated by the magnetic coil, the membrane potential also oscillated due to the neuron being driven by the high frequency stimuli. However, stimulation did not cause a dramatic depolarization or hyperpolarization of the membrane potential (Fig. 11a). The fast influx of the sodium current was interrupted and diminished during the high frequency coil stimulation (Fig. 11b). Coil stimulation prevented the activation of the sodium channels (m decreased from 0.95 to 0.2, Fig. 11d), and prevented sufficient de-inactivation of the sodium channels (h decreased from 0.4 to 0.2, Fig. 11e). Since the conductivity of the sodium channel is defined by m^3^h^32^, this result suggests that sodium channel conductance was reduced under magnetic stimulation, preventing the ignition of an action potential in the low-activity neurons. In the meantime, the potassium channels (n = 0.5, Fig. 11f) were not able to be activated, leading to a diminished outward potassium current (Fig. 11c).

**Figure 12:**
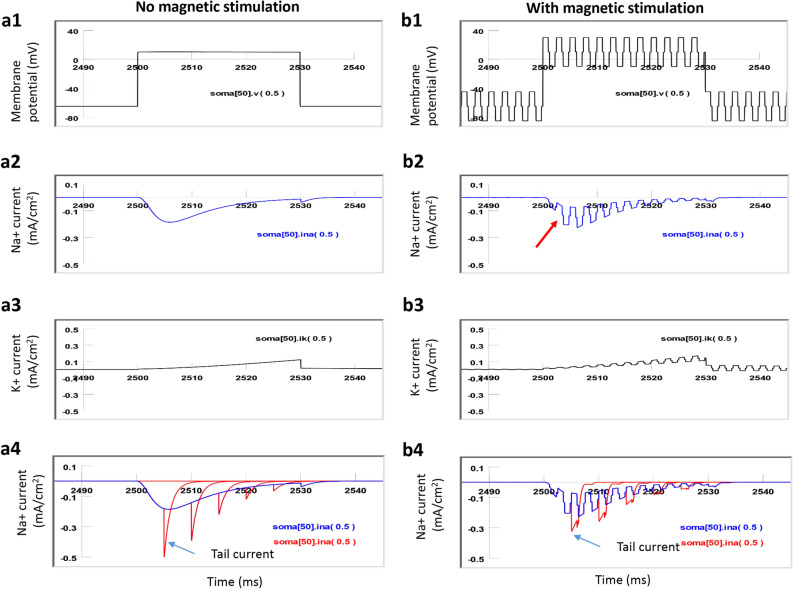
Sodium and potassium currents in a voltage clamp experiment (NEURON simulation). (**a1**) Without magnetic stimulation, the membrane potential was clamped from − 65 to 10 mV for 30 ms. (**a2**) Fast inward current (INa). (**a3**) Slow outward (IK). (**a4**) The sodium “tail currents” (red traces) were produced by varying the duration of clamping steps (from 5 to 30 ms, with 5 ms increments). The blue trace demonstrated the INa for a 30 ms clamping period. (**b1**) With a 400 Hz magnetic stimulation, oscillation in the membrane potential occurred. (**b2**) Fast inward current (INa). (**b3**) Slow outward current (IK). (**b4**) The amplitude of the sodium “tail currents” (red traces) was significantly decreased during magnetic stimulation.

To directly measure the sodium channel conductance, we performed a voltage clamp experiment using the model neuron (Fig. 12). The membrane potential in the middle of the soma (Soma^50^) was clamped from − 65 to 10 mV for 30 ms (Fig. 12a1), which led to a fast inward sodium current (INa, Fig. 12a2), followed by a delayed outward potassium current (IK, Fig. 12a3). When the high frequency (400 Hz) magnetic field was applied to the modeled neuron via the micro-coil, it caused a quick fluctuation of membrane potential that was superimposed on the clamped voltage (10 mV, Fig. 12b1). This led to a disruption of the quick rising phase in the sodium current (INa) and a reduction of the overall INa (Fig. 12b2). It also caused a fluctuation in the IK (Fig. 12b3), which may affect the shape of action potentials. By varying the duration of holding potentials (5–30 ms, with 5 ms increments), we recorded the sodium “tail currents” (Fig. 12a4). During magnetic stimulation, we observed a significant decrease in the amplitude of the sodium tail currents (Fig. 12b4), suggesting a significant decrease in the sodium channel conductance^32^. This voltage clamp experiment further confirmed that the magnetic field impaired normal channel dynamics by reducing the amount of inward sodium current and decreasing sodium conductance.

In summary, the state-dependent inhibition of single neuron activity is mediated by the differential modulation of the ion channel dynamics in the high-active neurons vs. low-active neurons. This modulation is caused by the oscillation of membrane potential in the high frequency magnetic field. While ion channels in the high-active neurons are insensitive to the magnetic stimulation, dynamics of ion channels in the low-active neurons are significantly affected. It caused insufficient sodium channel activation and de-inactivation, and decreased sodium conductance, leading to the failure of initiating and sustaining the action potentials in these neurons.

## Discussion

### State-dependent neural inhibition

The outcome of electrical and magnetic stimulation has been reported to be state-dependent at the behavioral level. Animals under these different states have distinct responses to neural stimulation with electric current^33^. Considerable changes have been observed in functional connectivity and correlated activity between the awake state and anesthesia both in monkeys^34^ and rodents^35^. This paper sought to understand how individual neurons respond to magnetic stimulation in a state-dependent manner.

We reported that the outcome of magnetic stimulation was dependent on the activation state of the target neurons. Specifically, the neurons at a lower excitability state (low firing rate) were more prone to inhibition by the high frequency coil stimulation. These action potentials could be spontaneous (Figs. 4, 5), or better controlled by depolarization current steps (Fig. 6) or short pulses of fixed frequency (Fig. 7). These protocols mimic a variety of excitatory inputs to the neuron with rhythmic or tonic synaptic inputs. Considering neural activity is dynamic, it is reasonable to speculate that the same neuron could have varied sensitivity to the same magnetic field. These results also suggest that neurons with less excitatory synaptic input are also more prone to inhibition. Therefore, variability in synaptic drive renders individual neurons variable to magnetic stimulation.

### Ion channel mechanisms of state-dependent magnetic inhibition

Previous works with high frequency electric stimulation for neural inhibition have reported that the behavior of ion channels largely depended on the waveforms used in these studies^36^. For example, in high frequency stimulation using monophasic electric pulses, the unidirectional electric current was shown to depolarize or hyperpolarize the membrane^18^. Excessive depolarization could lead to “depolarization blockage,” which inactivated sodium channels and impaired the mechanisms of neuronal firing^30^. In some chronic applications, biphasic stimulation with electric field was used for neural blockage in peripheral nerves^37,38^, since it caused less tissue damage when compared to monophasic stimulation due to the neutralization properties of electrochemical reactions^36^. Since biphasic electric currents can depolarize and hyperpolarize the nerve membrane alternatively, it cannot be assumed that the blockage is due to either membrane depolarization or hyperpolarization.

In our work with magnetic stimulation, we confirmed, via biophysical modeling and direct measurement, that the induced electric field is biphasic. Intracellular recording confirmed that the change in membrane potential was insignificant during high frequency magnetic stimulation. Instead, we observed a high frequency oscillation in the membrane potential (Figs. 4, 5, 6, 7). Therefore, the neurons were unlikely to be inhibited by the depolarization blockage and its associated ion channel changes.

Because experimental studies have been limited by techniques and methods available at times, modeling studies provide important insights into biophysical aspects of miniature coil stimulation. Specifically, we used multi-compartment NEURON modeling to simulate the effects of high frequency magnetic stimulation on a single *Aplysia* neuron. We observed that a high frequency magnetic field inhibited the neuron in a low activation state but spared the neuron in a high activation state (Fig. 9). Magnetic stimulation in the highly active neuron caused limited changes in the ion channel dynamics of the neuron (Fig. 10). In contrast, magnetic stimulation introduced significant changes in the low-activity neuron, including insufficient sodium channel activation and de-inactivation, and insufficient potassium channel activation. Voltage clamp experiments, by measuring the “tail currents,” confirmed that sodium channel conductance was significantly decreased by the magnetic stimulation (Fig. 12b4). These combined ion channel mechanisms led to the failure of sustaining the somatic action potentials in the low-activity neuron (Fig. 11). Previously, it was found that low frequency (15 or 50 Hz) magnetic stimulation was able to alter sodium and potassium channel activation in the pyramidal neurons in the rat’s hippocampus^31^. It would be interesting to directly use patch clamp technology to validate our model prediction about the alterations of ion channel dynamics under micro-coil stimulation at the higher frequency band that we explored.

### Limitations of the NEURON model and future work

The NEURON model in this study was adapted from a published model for the *Aplysia* neuron^18^. By implementing a simple geometrical structure to represent a soma and a straight axon, this model was not perfect in replicating all the behavior of an *Aplysia* neuron. For example, the model did not include the synaptic connection with other neurons, which could affect outcomes of magnetic stimulation^39^. The Hodgkin-Huxley based ion channel mechanism did not include several important ion channels that might be important for neural excitability, such as the Ca^2+^ channels and A-type K^+^ channels^10^. The model also did not consider the potential ion accumulation induced by magnetic stimulation, which could accumulate in the extracellular space^40,41^ and alter neural excitability. These limitations can be addressed in future endeavors when biological data becomes available to support the next iteration of modeling.

When the *Aplysia* neural model was first proposed^18^ based on the H–H model^32^, it adapted most of the parameters that defined the ion channel dynamics (i.e., activating state variables m, h, and n), which were best defined at a relatively low temperature (6.3 °C,^32^). We, therefore, chose to run the model at this default temperature in the H–H model, so that the model neuron (Figs. 10, 11) could generate action potentials whose firing frequency can match those from the electrophysiological recording (1–12 Hz). Running the model at room temperature (20 °C) produced a higher firing frequency in the neuron than that observed in the electrophysiological experiments from the *Aplysia* neuron. However, under both temperatures, we observed similar “state-dependent” neural inhibition and ion channel dynamics. To perfectly match the model observation with experimental data at the same room temperature, we need to modify the temperature-sensitive parameters in our model. This requires performing voltage-clamp experiments and analyzing the activating state variables at room temperature.

During the computation of the induced electric field and its interaction with the modeled neuron, the extracellular electric field was computed without consideration of the tissue and its counter effect on the externally applied electric fields, which could introduce some computational errors^3,42^. Though we modeled the geometry of the miniature coil as an infinitely long cylinder for computational simplicity, a more accurate representation of the coil shape and electric field calculation will be needed. In addition, considerations of tissue inhomogeneity and anisotropy will also be necessary for a more accurate representation of the induced electric field^13,43^. This can be accomplished by using finite element models^44^ with additional work in the future. Regardless of these model limitations, the current model is sufficient to simulate state-dependent neural inhibition by the magnetic coil and, for the first time, allows us to understand the channel dynamics underlying this interesting phenomenon in neuromodulation by electromagnetic stimulation.

The in vitro observation of state-dependent magnetic inhibition shall be further validated using in vivo preparation, in which the miniature coil will be implanted inside, close to the buccal ganglion, for stimulation. Recording single neuron activity from the behaving animals is also feasible, by attaching a small suction electrode on the buccal ganglion surface, next to the soma^45^.

Finally, this work proposes that state-dependent neural inhibition by magnetic stimulation is associated with membrane potential changes and the associated ion channel dynamics. The quick, reversible inhibition of the neural activity by the miniature coil stimulation is indeed associated with the membrane oscillation in our experimental (Figs. 4, 5, 6, 7) and simulation (Fig. 9) results. Many other works have also provided experimental and theoretical evidence to support this notion (reviewed in^3^). However, the impact of the magnetic field is not limited to neurons and ion channel activation/deactivation^46^. Magnetic fields can alter an array of cellular physiology processes, such as cell proliferation^47^, microglial activation^48^, and the production of reactive oxygen species^49^, which can produce prolonged post-treatment effects in magnetic stimulation.

### Implications of state-dependent neural stimulation on basic cellular research

The discovery that state-dependency can be observed at the single cell level has several implications to basic research in neuromodulation with electric and magnetic stimulations.

First, this work enriches our understanding of the neural response to electric and magnetic fields, and its dependency on the cell’s intrinsic properties. Previous works have identified these important properties, including the morphological and electrical properties of a single neuron, the density of the neurons within a tissue, and ephaptic interactions between far-distance neurons^3^. The work presented here highlights the need to understand the modulatory effects of electric and magnetic stimulation in the context of individual neurons’ dynamic and excitatory states.

Second, since the level of neural excitability changes over time, it is worth monitoring these changes to optimize stimulation outcomes. For example, recreational use of drugs is commonly associated with an increased excitability of neurons^50^, and excitability may change during aging^51^ and in pathological conditions such as seizures^52^. Consequently, experimental protocols for neuromodulation with electromagnetic stimulation should consider matching or compensating for these dynamic changes at the cellular level.

Third, state-dependent responses of the neuron to electric and magnetic stimulation could also impact other functions of the neurons. For example, neuronal excitability plays significant roles in cell migration^53^, myelination processes^54^, post-translational modification of synaptic molecules ^55^, and the transcription of a large set of genes^56^. If electric or magnetic stimulation were to be used to control these processes by regulating the excitability of the neuron, one would expect to observe state-dependent outcomes in these neural functions.

### Implications of state-dependent stimulation to clinical neuromodulation with high frequency magnetic fields

Previously, it was found that high frequency magnetic stimulation can inhibit neuron activity, including axonal blockage^19^ and somatic inhibition^20^. These stimulation effects, referred to as ‘‘virtual lesions’’^4^, provided a method for the reversible blockage of neural function without structural brain lesions that cause permanent functional deficits.

Traditionally, high frequency signals were more widely used in electric stimulation than in magnetic stimulation, such as in deep brain stimulation (DBS)^57^ or peripheral nerve blockage^58^ with electrode. This is mainly due to the fact that it is technologically challenging to generate high frequency pulses with a large magnetic coil for TMS, for reasons such as energy-storage requirements and potential thermal effects caused by high frequency current in the large coil.

However, recent developments in repetitive transcranial magnetic stimulation (rTMS) have demonstrated the possibility of using a high frequency stimulus for magnetic stimulation. For example, a stimulation paradigm employing bursts of high frequency (50 Hz) rTMS^59^, known as theta-burst stimulation (TBS), significantly reduced motor cortical excitability when applied continuously. High frequency rTMS trains have demonstrated long-term anticonvulsant effects^60^ in some animal studies. High frequency rTMS could decrease epileptic spike frequency acutely^61^. High frequency stimulation with a miniature coil at 400 Hz is also effective in suppressing epileptiform activity in hippocampal slices in vitro^62^.

The intensity of the magnetic field generated by the miniature coil is 54.3 mT (Eq. 4). Although this value is significantly smaller than that used in clinical rTMS applications (several Tesla), the close positioning of the miniature coil to the targeted neuron ensured large gradients of the induced electric field for neural stimulation^22,63,64^.

This work provides strong evidence that the state of the neurons plays significant roles in magnetic stimulation. Although the work is from a model system of invertebrate, which does not allow us to speculate the outcomes of our stimulation protocols if applied to human neurons, it strongly supports some of the pioneer ideas in clinical TMS and DBS practices. Specifically, it highlights the importance of state-dependent stimulation in clinical settings using high frequency rTMS. It also highlights the importance of monitoring the activation state of the nervous system for the best outcomes in these clinical practices, which can be supported by the following applications.

First, pre-existing activity levels can modulate the stimulation intensity required to evoke an overt response. For example, it was found that higher pre-TMS activity predicts larger post-TMS responses^17^. It is, therefore, essential to understand pre-existing activity levels to predict the outcome of such stimulation procedures.

Second, since neural activity changes over time, it is essential to monitor the activity state of the targeted neurons and neural network during brain stimulation. Changes in neural activity can also be observed in hemodynamic signals for effective TMS^65^. Combined TMS with functional magnetic resonance imaging (fMRI) is powerful in revealing how different TMS intensities could induce different local and remote activation^66^. This combined approach with imaging technology could provide an empirical guide for the effective use of TMS in both clinical and experimental settings.

Third, it is essential to develop technology that can apply state-dependent brain stimulation. Electroencephalography (EEG) can be used to monitor the fluctuations of the brain state^67^. Real-time, multi-channel EEG data can be used to monitor the brain state online and modify stimulation parameters^68^ to apply state-dependent brain stimulation. EEG can also be used to design closed-loop, purpose-driven stimuli, to provide brain-state guided stimulation^69,70^.

Fourth, it is essential to develop technology that can precondition the state of the neural network to enhance the stimulation outcome^71^. For example, it is possible to use transcranial direct current stimulation (tDCS) to precondition the low frequency rTMS of the motor cortex, and this preconditioning reversed the effects of 1 Hz rTMS^72^.

Finally, it is possible to consider using pharmacological approaches to alter the excitability of the nervous system and maximize the clinical outcome of rTMS, as demonstrated in several reports. For example, when anticonvulsant phenytoin was administered, the magnetic field was more effective in decreasing audiogenic seizure severity in mice^73,74^. Similarly, bursts of high frequency rTMS, together with lorazepam, suppressed seizures in a rat kainate status epilepticus model^61^, with the combined method being more effective than rTMS alone. Future research should explore the possibility of improving complementary therapies by adjusting the excitability state of the nervous system.

## Methods

### Magnetic stimulation of the neuron

A multilayer surface mount inductor (100nH, MLG1005SR10JTD25, TDK U.S.A. Corporation, Uniondale, NY) was used as the miniature coil for neural stimulation (Fig. 1). The coil was coated with acrylate copolymer enamel (Revlon, New York)^75^ for electric isolation. An arbitrary function generator (AFG1022, Tektronix) was used to generate a stimulation signal. Monophasic square waves (50% duty cycle) of various frequencies were generated and delivered into the power amplifier. The signal triggered large current pulses through a 1000 W power amplifier (Pyramid PB 717X 2 channel, Pyramid Car Audio, Brooklyn, NY, 11204). The output of the power amplifier was connected to the two leads of the miniature coil to generate time-varying magnetic fields. The amplifier was powered by a triple-channel DC power supply (2231A-30-3, Keithley). The power amplifier works as a constant voltage source. For frequencies higher than 5 Hz, voltage across the coil also maintained square pulses. Square waves of various frequencies (5–500 Hz) were delivered to the power amplifier for the stimulation. The voltage across the two coil ends was measured and recorded. Voltage changes around the coil were measured in a petri dish filled with *Aplysia* saline (Fig. 2).

The impedance of the coil was measured at the beginning and end of each experiment to test its connectivity. Potential leakage of the coating coverage was also tested by measuring the impedance of the coil to the ground. If present, this leaking current could generate an extremely large level of noise. The local temperature around the coil was measured with a thermocouple, which was connected to a digital thermometer (HH11B, Omega Engineering, Norwalk, CT) to display the temperature with 0.1 °C resolution. Throughout the experiments, we did not observe noticeable temperature increases.

To illustrate the structure of the coil (Fig. 1b), we removed the ceramic core and epoxy coating following a published protocol^62^, using 40% liquid hydrofluoric acid at room temperature for 48 h, followed by 10 N HCL for 1 h.

### In vitro electrophysiology and miniature coil stimulation

*Aplysia californica* (100–150 g) were obtained from Marinus Scientific (Newport Beach, CA) and kept in artificial seawater at room temperature (20 ± 1 °C). Animals were anesthetized by an injection of isotonic MgCl_2_ (50% of body weight). The buccal ganglion was dissected and immersed in an *Aplysia* saline solution (pH 7.4), which contained 460 mM NaCl, 55 mM MgCl_2_.H_2_O, 11 mM CaCl_2_.2H_2_O, 10 mM KCl, and 10 mM Hepes. The buccal ganglion was completely de-sheathed to expose the cell bodies. The preparation was put into a 4 °C refrigerator for 1 h to allow the neurons to recover from dissection before the electrophysiology experiments were performed at room temperature.

The intracellular electrodes were made by pulling single-barreled capillary glasses using a Flaming-Brown micropipette puller (P-30, Sutter Instrument). The pulling protocol was adjusted so that the tip of the electrode was sharp for cell penetration. Sharp electrodes were backfilled with 3 M potassium acetate before use. For intracellular recording, intracellular signals were amplified using a DC-coupled amplifier (model 1600, A-M systems). DC offset was eliminated, and the bridge was balanced before inserting the electrode into the cell body for stimulation and recording. Large jaw motor neurons (i.e., B3, B6, B9) on the caudal side of the buccal ganglion were recorded. These neurons have similar physiological properties and functions. They each send axons to the jaw closure muscle I1/I3 via the buccal nerve II (BN2)^27–29,76^. To avoid any post-stimulation effects, a single neuron from each animal was magnetically stimulated and recorded. To elicit action potentials, depolarization currents of various intensities were injected into the neuron. To precisely control the frequency of firing of the recorded neurons, an isolated pulse stimulator (model 2100, A-M systems) was connected to the 1600 amplifier to deliver short pulses at various frequencies. Intracellular recordings were digitized (25 kHz) by a CED 1401, then recorded and analyzed by Spike 2 software (version 7.2, Cambridge Electronic Design Limited). For magnetic stimulation, the miniature coil was positioned by a micromanipulator above the buccal ganglion. The coil was oriented so that its induced electric field was parallel to the ganglion—BN2 axial (Fig. 3) to produce effective stimulation^25,26^ of the recorded motor neurons.

### Calculation of magnetic field generated by a miniature coil

When an electric pulse was delivered to the coil, it generated a magnetic field around the coil. The voltage across the coil was equal to the voltage drop due to the coil resistance and inductive impedance,

1 $$ V = IR + L\frac{dI}{{dt}} $$

where I was the current in the coil, R was the coil resistance, and L was the inductance of the coil.

For the rising phase of the pulse, solution of the coil current was

2 $$ I = \frac{V}{R}\left( {1 - e^{{ - \frac{tR}{L}}} } \right) $$

Therefore, the coil current was zero at the beginning of the pulse and increased exponentially to a plateau value (V/R). For the falling phase of the pulse, the coil current was

3 $$ I = \frac{V}{R}e^{{ - \frac{tR}{L}}} $$

Therefore, the coil current decayed exponentially in the falling phase, from maximum (V/R) to zero.

For a coil with a flowing current (I) inside, the magnetic field was calculated by

4 $$ B = \mu_{0} \frac{NI}{l} = \frac{{\mu_{0} NV}}{Rl}\left( {1 - e^{{ - \frac{tR}{L}}} } \right) $$

for the rising phase, or

5 $$ B = \mu_{0} \frac{NI}{l} = \frac{{\mu_{0} NV}}{Rl}e^{{ - \frac{tR}{L}}} $$

for the falling phase, where N was the loop of the coil, $l$ was the length of the coil, and *μ*_0_ = 4π × 10^−7^ H/m was the vacuum permeability.

### Calculation of the induced electric field by a magnetic coil

A magnetic field generated by the coil could also be calculated by Faraday’s law of induction,

6 $$ \varepsilon = - \frac{{d\phi_{B} }}{dt} $$

where ɛ was the electromotive force (EMF) and $\Phi$_B_ was the magnetic flux. It could also be written in an integral form (Kelvin–Stokes theorem),

7 $$ \oint {\mathop{E}\limits^{\rightharpoonup} \cdot d\mathop{l}\limits^{\rightharpoonup} = - \iint {\frac{{\partial \mathop{B}\limits^{\rightharpoonup} }}{\partial t}}} \cdot d\mathop{A}\limits^{\rightharpoonup} $$

where $\mathop{B}\limits^{\rightharpoonup}$ was the magnetic field inside the coil, $\mathop{E}\limits^{\rightharpoonup}$ was the induced electric field, $d\mathop{l}\limits^{\rightharpoonup}$ was an infinitesimal vector element or the path element, and $d\mathop{A}\limits^{\rightharpoonup}$ was an infinitesimal vector element of the area considered.

Figure 8 illustrates the cylinder system, whose center was overlapping with the center of the coil. For a point A (r, θ) in this system, from Eq. (7),

8 $$ E_{\theta } = - \frac{{R_{c}^{2} }}{2r}\frac{\partial B}{{\partial t}}\;\;\;\left( {{\text{r }} > {\text{ R}}_{{\text{c}}} } \right) $$

9 $$ E_{r} = 0\;\;\;\left( {{\text{r }} > {\text{ R}}_{{\text{c}}} } \right) $$

Here, $R_{c}$ was the radius of the coil, and r was the distance between an arbitrary point to the center of the coil.

Combining Eqs. (4) and (8), the induced electric field (outside the coil) during the rising phase was

10 $$ E_{\theta } = - \frac{{R_{c}^{2} }}{2r}\frac{{V\mu_{0} N}}{Ll}e^{{ - \frac{tR}{L}}} $$

Similar analysis was applied to the falling phase by combining Eqs. (5) and (8),

11 $$ E_{\theta } = \frac{{R_{c}^{2} }}{2r}\frac{{V\mu_{0} N}}{Ll}e^{{ - \frac{tR}{L}}} $$

Therefore, the induced electric field was the largest at the rising and falling phases (with opposite sign), falling to zero with time following a relaxation course. The electric field needs to be large enough at the on and off phases for effective stimulation.

This waveform was experimentally measured by putting a recording electrode close to the coil in a petri dish filled with *Aplysia* saline (Fig. 2). The shape of the induced electric field was biphasic in shape, and exponentially decayed after reaching the peaks. When the duration of the square pulse was long (> 2 ms), the waveform for each of the pulses was identical, and was independent of stimulation frequency.

To incorporate the biophysical model of the miniature coil with the multi-compartment model of the *Aplysia* neuron, we applied the induced electric field to the model neuron (Fig. 3). It is well-accepted that the gradients of the electric field along the soma-axon axis define the location and speed of depolarization or hyperpolarization by extracellular stimulation^22,63^. Therefore, we calculated the electric potential along the axon, in the x-axial direction.

In Fig. 8, the coil-induced electric field could be expressed on a Cartesian basis using matrix transformation,

12 $$ E(x,y) = \left[ {\begin{array}{*{20}c} {\cos \theta } & { - \sin \theta } \\ {\sin \theta } & {\cos \theta } \\ \end{array} } \right]\left[ {\begin{array}{*{20}c} {E_{r} } \\ {E_{\theta } } \\ \end{array} } \right] $$

where $$\mathrm{sin}\theta =\frac{y}{r}$$ , $$\mathrm{cos}\theta =\frac{x}{r}$$ , and $$r = (x^{2} + y^{2} )^{1/2}$$.

For the rising phase of the pulse, using Eqs. (10) and (12),

13 $$ E_{x} = - \frac{{V\mu_{0} NR_{c}^{2} }}{2Ll}\frac{y}{{x^{2} + y^{2} }}e^{{ - \frac{tR}{L}}} $$

14 $$ E_{y} = \frac{{V\mu_{0} NR_{c}^{2} }}{2Ll}\frac{x}{{x^{2} + y^{2} }}e^{{ - \frac{tR}{L}}} $$

For the falling phase of the pulse, using Eqs. (11) and (12),

15 $$ E_{x} = \frac{{V\mu_{0} NR_{c}^{2} }}{2Ll}\frac{y}{{x^{2} + y^{2} }}e^{{ - \frac{tR}{L}}} $$

16 $$ E_{y} = - \frac{{V\mu_{0} NR_{c}^{2} }}{2Ll}\frac{x}{{x^{2} + y^{2} }}e^{{ - \frac{tR}{L}}} $$

For the rising phase of the pulse, the electric potential distribution along the axon is

17 $$ V\left( x \right) = \int E_{x} \left( x \right)dx = - \frac{{V\mu_{0} NR_{c}^{2} }}{2Ll}{\text{atan}}\left( \frac{x}{y} \right)e^{{ - \frac{tR}{L}}} $$

For the falling phase of the pulse, the electric potential distribution along the axon is

18 $$ V\left( x \right) = \int E_{x} \left( x \right)dx = \frac{{V\mu_{0} NR_{c}^{2} }}{2Ll}{\text{atan}}\left( \frac{x}{y} \right)e^{{ - \frac{tR}{L}}} $$

We used the parameters of the miniature coil that were provided by the manufacturer for the computation. This included the length (l = 0.5 mm), resistance (R = 2 Ω), and inductance (L = 100 nH) of the inductor. Since each loop of the coil was a 1 mm × 0.5 mm rectangle shape (Fig. 1B), the coil was modeled as a circle with Rc = 0.25 mm. When 20 V square waves (-10 V/10 V peak to peak) were applied, the output of the coil was V = 2.16 V.

### NEURON modeling of an *Aplysia* neuron

Effects of coil stimulation on a single neuron were tested with a published multi-compartment soma-axon model of an *Aplysia* neuron^18^ using the NEURON simulation environment package^77^. The model was modified to simulate neuron activity under magnetic stimulation with a miniature coil^20^. Briefly, the modeled *Aplysia* neuron contained a spherical soma and a cylindrical axon. The diameter of the soma (D) was 200 µm, matching the size of a large motor neuron (i.e., B3, B6, and B9) in the buccal ganglion (Fig. 3). The soma was divided into 100 (N, i = 0–99) segments along its soma-axon axis. Each segment was 2 µm in length and was in the shape of a cylinder-disk. The soma tip segment (i = 0) was set to be 1 µm in diameter. The diameters of the rest of the soma disks, D(i) (i = 1–99), were computed as a function of its distance to the center of the soma (Fig. 8).

19 $$ D\left( i \right) = \sqrt {\left( \frac{D}{2} \right)^{2} - \left[ {\left( {\frac{N}{2} - i} \right)\frac{D}{N}} \right]^{2} } $$

The axon cylinder was 15 μm in diameter and 20,000 μm in length and was divided into 200 segments of equal length.

The Hodgkin-Huxley (H–H) type of fast sodium, slow potassium, and leakage channels in the membrane were inserted into each compartment of the modeled neuron^32^. The ionic current at the n-th segment of the neuron was expressed as

20 $$ I = g_{Na} m^{3} h\left( {V - V_{Na} } \right) + g_{k} n^{4} \left( {V - V_{k} } \right) + g_{L} \left( {V - V_{L} } \right) $$

where V_Na_, V_K_, and V_L_ were the equilibrium membrane potentials for sodium, potassium, and leakage channels, respectively. g_Na_, g_k_, and g_L_ were the maximal conductances of Na, K, and leakage channels, respectively (Table 1). V is the membrane potential in the n-th segment. The parameters m and h represented the activation and inactivation of the sodium channels, respectively, whereas n represented the activation of potassium channels. The evolution equations for variables m, h, and n were

21 $$ \frac{dm}{{dt}} = \alpha_{m} \left( {1 - m} \right) - \beta_{m} m $$

22 $$ \frac{dh}{{dt}} = \alpha_{h} \left( {1 - h} \right) - \beta_{h} h $$

23 $$ \frac{dn}{{dt}} = \alpha_{n} \left( {1 - n} \right) - \beta_{n} n $$

where

$$ \begin{array}{*{20}c} {\alpha_{m} = \frac{{0.1\left( {V + 40} \right)}}{{1 - {\text{exp}}\left( { - 0.1\left( {V + 40} \right)} \right)}}} & {\beta_{m} = 4{\text{exp}}\left( { - \frac{V + 65}{{18}}} \right)} \\ \end{array} $$

$$ \begin{array}{*{20}c} {\alpha_{h} = 0.07{\text{exp}}\left( { - 0.05\left( {V + 65} \right)} \right)} & {\beta_{h} = \frac{1}{{1 + {\text{exp}}\left( { - 0.1\left( {V + 35} \right)} \right)}}} \\ \end{array} $$

$$ \begin{array}{*{20}c} {\alpha_{n} = \frac{{0.01\left( {V + 55} \right)}}{{1 - {\text{exp}}\left( { - 0.1\left( {V + 55} \right)} \right)}}} & {\beta_{n} = 0.125{\text{exp}}\left( { - \frac{V + 65}{{80}}} \right)} \\ \end{array} $$

**Table 1:** Electric parameters of the NEURON model for an *Aplysia* neuron that contains a soma and an axon.

Electrical parameter	Value
Membrane capacitance (*C*_m_)	1 μF/cm^2^
**Fast Na**^**+**^** channels**	
Max. sodium conductance (g_Na__) in the soma	0.024 S/cm^2^
Max. sodium conductance (g_Na__) in the axon	0.12 S/cm^2^
Reversal potential (*E*_Na_)	50 mV
**Slow K**^**+**^** channels**	
Max. potassium conductance (g_K__) in the soma	0.0072 S/cm^2^
Max. potassium conductance (g_K__) in the axon	0.036 S/cm^2^
Reversal potential (*E*_K_)	− 77 mV
**Leakage channels**	
Conductance (*g*_L_)	0.00028 S/cm^2^
Reversal potential (*E*_L_)	− 65 mV

t: environmental temperature in degrees Celsius.

v: membrane potential of a neural segment in mV.

Detailed electrical parameters of the modeled soma and axon (Table 1) were adapted from the published model of the *Aplysia* buccal neuron^18^. To simulate the lower densities of Na^+^ and K^+^ channels in the soma compared to the axon, the maximal conductance of Na^+^ and K^+^ channels in the soma were set to be 1/5 of those in the axon^18^. The time constants of the Na^+^ and K^+^ channels were increased by linear scaling factors based on the ratios of the time constants of the Hodgkin-Huxley model to the time constants of *Aplysia* sensory neurons^78^.

During NEURON simulation, the electric voltage induced by the miniature coil (Eqs. 17 and 18) was applied to the modeled NEURON using the “play” function^44^. To simplify the calculation, we set the center of the modeled soma as (0,0), and a point A (X, 0) was defined as the location of an arbitrary point (A) on the modeled cell (Fig. 8). The center of the coil was defined at (x_coil_, y_coil_). Therefore, in Eqs. (17) and (18), $x=X-{x}_{coil}$ and $y={y}_{coil}$. The model was set to run at the default temperature (6.3 °C) as first introduced in the H–H model^32^ and Lu model^18^. We also ran the model at room temperature (20 °C). Simulation with both temperatures produced qualitatively similar results. Since precise modeling of the exponential rise or decay of the induced electric voltage was computationally challenging, we used biphasic, short pulses with alternating direction to represent the induced electric field. The duration of the pulse was 1 ms, as observed in our actual measurements (Fig. 2).

### Statistics

The inhibitory effects of coil stimulation on neurons that fire at different frequencies were compared with Chi-square analysis using SigmaStat 3.01a (Systat Software, Inc.).

## Data Availability

All data generated or analyzed during this study are included in this published article.
